# Spontaneous Breathing Pattern as Respiratory Functional Outcome in Children with Spinal Muscular Atrophy (SMA)

**DOI:** 10.1371/journal.pone.0165818

**Published:** 2016-11-07

**Authors:** A. LoMauro, A. Aliverti, C. Mastella, M. T. Arnoldi, P. Banfi, G. Baranello

**Affiliations:** 1 Dipartimento di Elettronica, Informazione e Bioingegneria; Politecnico di Milano, Italy; 2 S.A.PRE., Ospedale Policlinico Maggiore Mangiagalli, and Regina Elena Foundation, Milan, Italy; 3 Developmental Neurology Unit, Carlo Besta Neurological Research Institute Foundation, Milan, Italy; 4 Pulmonary Rehabilitation Fondazione Don Carlo Gnocchi, Milan, Italy; Iowa State University, UNITED STATES

## Abstract

**Introduction:**

SMA is characterised by progressive motor and respiratory muscle weakness. We aimed to verify if in SMA children 1)each form is characterized by specific ventilatory and thoraco-abdominal pattern(VTAp) during quiet breathing(QB); 2)VTAp is affected by salbutamol therapy, currently suggested as standard treatment, or by the natural history(NH) of SMA; 3)the severity of global motor impairment linearly correlates with VTAp.

**Materials and methods:**

VTAp was analysed on 32 SMA type I (SMA1,the most severe form), 51 type II (SMA2,the moderate), 8 type III (SMA3,the mildest) and 20 healthy (HC) using opto-electronic plethysmography. Spirometry, cough and motor function were measured in a subgroup of patients.

**Results:**

In SMA1, a normal ventilation is obtained in supine position by rapid and shallow breathing with paradoxical ribcage motion. In SMA2, ventilation is within a normal range in seated position due to an increased respiratory rate(p<0.05) with reduced tidal volume(p<0.05) secondary to a poor contribution of pulmonary ribcage(%ΔV_RC,P_, p<0.001). Salbutamol therapy had no effect on VTAp during QB(p>0.05) while tachypnea occurred in type I NH. A linear correlation(p<0.001) was found between motor function scales and VTAp.

**Conclusion:**

A negative or reduced %ΔV_RC,P_, indicative of ribcage muscle weakness, is a distinctive feature of SMA1 and SMA2 since infancy. Its quantitative assessment represents a non-invasive, non-volitional index that can be obtained in all children, even uncollaborative, and provides useful information on the action of ribcage muscles that are known to be affected by the disease.Low values of motor function scales indicate impairment of motor but also of respiratory function.

## Introduction

Spinal Muscular Atrophy (SMA) is characterized by degeneration of α motoneurons in the spinal cord, resulting in progressive motor and respiratory muscle weakness and paralysis[1–5]. According to severity and age of onset, clinical phenotypes have been grouped into the most severe type I (SMA1), the moderate type II (SMA2) and the mildest type III (SMA3) forms[6–8].

Respiratory problems are the major causes of hospitalization, morbidity and mortality in SMA children[9]. Over the last decade, proactive management of respiratory complications has rapidly increased, thus improving survival and quality of life.[10–14] Additionally, an increasing number of potential therapeutic strategies are entering or have already entered clinical phases[5,15,16]. Specific respiratory outcome measures are therefore needed to objectively evaluate the effects of interventions in all SMA children.

Respiratory function can be assessed by invasive and non-invasive techniques[17–21]. Invasive measurements are generally not well tolerated by children, and non-invasive techniques should be considered. These include the measure of spirometric parameters, peak cough flow (PCF)[22], ventilatory pattern and thoraco-abdominal contribution during spontaneous quiet breathing at rest (QB).

Since the early childhood, in SMA1 and SMA2 spirometric indexes are reduced and show a relatively slow rate of decline, eventually worsened by scoliosis[23–28]. Cough is inefficient and requires assistance since the first years of life[29–31]. Both spirometry and PCF measurement are volitional tests requiring high collaboration and therefore being consistently performed since the age when the child understands the operation. On the other hand, non-volitional measurement during QB is feasible in almost all patients, even those uncooperative.

SMA children are at risk of hypoventilation, and ventilatory support is needed to reverse the resulting reduced arterial oxyhemoglobin saturation and microatelectasis.[14] Rapid and shallow breathing ensues in awake SMA2, hypothesized to be a respiratory strategy these children adopt to minimize their work of breathing[23].

None of the above techniques or parameters provides specific information on inspiratory ribcage muscles (iRCM) status. This would be useful, as in SMA the phrenic motoneurons, and consequently the diaphragm, are preserved[32], while progressive weakness affects iRCM[9].

The assessment of thoraco-abdominal contribution during QB allows to selectively study the involvement of the different respiratory muscle groups. Thoraco-abdominal asynchrony and reduced ribcage expansion have been reported in SMA1 and SMA2 children but only lying supine[17–19]. In this position, the diaphragm lengthens, becoming the leading respiratory muscle with increased tension development. In supine position, therefore, a possible deficit of iRCM can be masked by the action of the diaphragm. For this reason, it would be of clinical interest to study these children also in seated position when iRCM and the diaphragm almost equally contribute to tidal volume[33]

We therefore hypothesized that 1) the three forms of SMA are characterized by different and specific ventilatory and thoraco-abdominal patterns during QB and 2) the rib cage contribution to tidal volume provides specific information on iRCM, and is affected by posture. To verify this, the respiratory pattern of young children affected by the three forms of SMA was separately described in seated and supine position and compared with healthy children. In addition to these two main hypotheses, we also verified if 3) the breathing pattern is affected by salbutamol therapy, being proposed to have beneficial effect on motor function and spirometry in SMA by increasing the survival motorneuron transcript levels in leucocytes, and currently suggested as standard supportive treatment for children with SMA in many neuromuscular centres[34–36]; and 4) respiratory outcomes are linearly correlated with the severity of global motor impairment, assessed by functional motor scales, as already reported for adult SMA patients[37]. Finally, as parents of SMA1 infants are called to choose between leaving the pure natural history of the disease to take its course or to use respiratory supports, we also investigated if the breathing pattern differs between these two groups of SMA1 infants[10,11,38,39].

## Materials and Methods

### Subjects and clinical features

This is a prospective cross-sectional study, approved by the Research Ethics Board of the Carlo Besta Neurological Research Institute (registration number: CE: 20/2014), on SMA children enrolled according to the following criteria: genetically proven diagnosis of SMA; age<8 years; absence of previous spinal surgery, acute respiratory failure, airway infections and 24-hours mechanical ventilation dependence.

In the patient population, cough assistance device was routinely used by 57% of SMA1 and 90% of SMA2 children, nocturnal ventilation respectively by 40% (two SMA1 patients were tracheostomized) and 29%, spinal bracing by 50% and 82%, and the 41% of SMA2 were on salbutamol at a stable dosage from at least 12 months. Eleven (34%) SMA1 and ten (19.5%) SMA2 children had at least one hospitalization for acute respiratory illness within the previous year (or since birth for infants) before the acquisition.

Parents of 10 SMA1 infants refused any respiratory support.

A control group of healthy children was also included.

All parents signed a written informed consent.

### Motor and lung function assessment

Global motor function was assessed by applying the 16-item scale Children’s Hospital of Philadelphia Infant Test for Neuromuscular Disorders (CHOP INTEND) scale[40,41] for SMA1, while the 33-items Hammersmith functional motor scale expanded (HFMSE)[42] and the 9-items Upper Limb Module (ULM) were used in SMA2 and SMA3. The HFMSE assesses motor function (e.g. lying, rolling, sitting, crawling, attaining standing, walking, running and jumping) in order of progressive difficulty, with higher values showing higher function abilities. It incorporates 13 relevant items to eliminate the ‘‘ceiling” effect of the original scale when applied to ambulant SMA patients. The 9 items of ULM were specifically developed to assess upper limb function in non-ambulant SMA patients, including young low functioning children [42]. The total score is calculated by summing the scores of the individual items and it can range from 0 (all activities failed) to 64, 66 and 18 (all activities achieved unaided), for the CHOP INTEND, HFMSE and ULM respectively.Forced vital capacity (FVC), forced expiratory volume in one second (FEV_1_), their ratio (FEV_1_/FVC) and peak cough flow (PCF) were measured (Pony FX PNT, Cosmed, Rome-Italy) when children were able to reasonably understand and follow instructions. The criteria used to determine if the spirometry tests were performed to an acceptable degree followed the Thoracic Society/European Respiratory Society Statement[43]. Predicted spirometric values were calculated by applying the global lungs initiative equations for spirometry[44,45] while predicted values of PCF according to Bianchi and Baiardi[46]. The numerosity of the percentage values was reduced compared to absolute values because there are no prediction values for children younger than the age of 4.

### Breathing pattern and thoraco-abdominal contribution during QB

**Fig 1:**
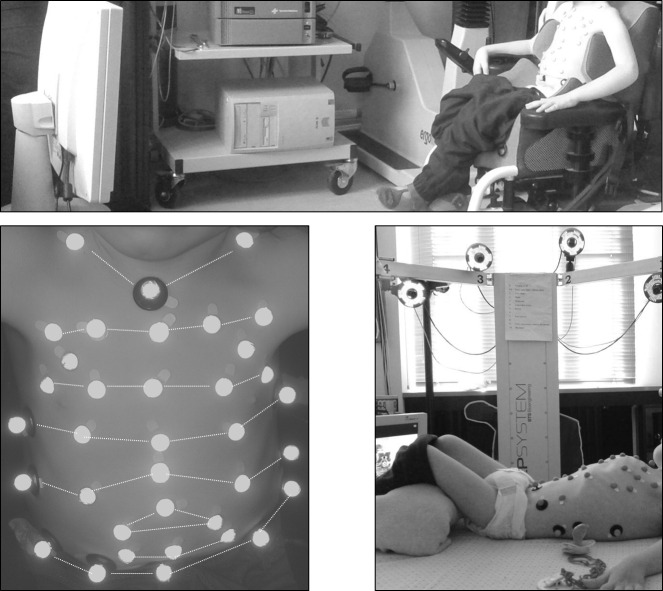
Experimental set-up for the analysis of chest wall volumes *via* opto-electronic plethysmography of a SMA child in seated (top) and supine (bottom right) positions while watching a cartoon. SMA3 and HC could sit without support and therefore 89 and 52 markers were respectively used in seated and supine position[44,45,48,50]. SMA2 children were all wheelchair bound and in both postures a new model was used with five horizontal rows of five markers, three rows of three markers with two additional markers making a total of 36 (bottom left). SMA1 were analyzed only in supine position, using the 36 model as well.

The volume variations of the chest wall (ΔV_CW_) and its compartments, namely pulmonary ribcage (ΔV_RC,P_), abdominal ribcage (ΔV_RC,A_) and abdomen (ΔV_AB_) were measured by opto-electronic plethysmography (OEP, BTS, Milan, Italy)[47,48] during at least 5 minutes of spontaneous QB before in seated and then other 5 minutes in supine position with the child awake (Brazelton stage 4)[49] while watching a cartoon (Fig 1). If the child was drowsy or slept or cried all the time during the acquisition, and therefore intervals of QB were impossible to be identified, he/she was excluded from the study.

The following parameters were calculated during inspiration of a 40-seconds period of QB: respiratory rate (RR), tidal volume (V_T_), minute ventilation (V´_E_ = RR*V_T_), rapid and shallow breathing index (RSBi = RR/V_T_), the percentage contribution of each compartment to V_T_ (%ΔV_RC,P_, index of iRCM action; %ΔV_RC,A_, index of the expansion of the appositional zone of the diaphragm and %ΔV_AB_, index of diaphragmatic action) and the phase shift angle between ΔV_RC,P_ and ΔV_AB_ (ϕ_TA_). V_T_ was calculated as the difference between body-weight normalized ΔV_CW_ and the total anatomic dead space body-weight normalized.[50]. The accuracy of OEP system during quiet breathing has been previously tested by simultaneous measurements with a spirometer in healthy adults[47,51], newborns[52] and infants[53]. In all these studies, the discrepancy between the two measurements was always <4%. The intraclass correlation coefficient values of OEP was shown to be higher than 0.75 while the coefficient of variation less than 10%. These parameters indicate that OEP presents adequate intra- and inter-rater reliability[54].

### Statistical analysis

Differences in anthropometric data, respiratory parameters and motor function were tested applying a one-way Analysis of Variance(ANOVA) with disease as independent factor. When normality test failed, a Kruskal-Wallis one-way ANOVA on Ranks was performed. Post-hoc tests based on Holm–Sidak and Dunn’s methods were respectively used for parametric and non-parametric ANOVA.

The same analysis was performed, considering medication or the family choice as independent factor, to test if motor and respiratory function of SMA2 under salbutamol therapy were different than not-treated children or if differences were present between SMA1 infants whose parents had chosen to follow the natural history of SMA or to support ventilation.

The least-square linear regression analysis was performed to verify if motor functional scales linearly correlated with the respiratory function parameters significantly affected by the disease.

Data are expressed as median and interquartile range (IQR). Significance was determined by p<0.05.

## Results

### Anthropometric, spirometric and cough data

**Fig 2:**
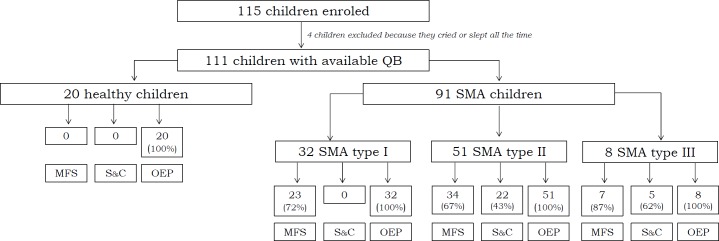
Flow diagram of recruited children and functional tests. QB: quiet breathing; MFS: motor functional scale; S_&_C: spirometry and cough tests; OEP: opto-electronic plethysmography

One-hundred-fifteen children were enrolled and data on 111 reported. Four children were excluded because it was not possible to obtain at least 40 seconds of QB (Fig 2).

Age was similar between SMA2, SMA3 and healthy (HC, median: 62.4, IQR: 52.5–72.0 months) children, while SMA1 were younger and characterized by lower weight (HC, 18.0,15.5–22.0 Kg) and height (HC, 114.5, 110.0–120.0 cm), as shown in Table 1.

**Table 1:** median and interquartile range (IQR) of anthropometric, motor function, spirometric and cough data in SMA types I (SMA1), II (SMA2) and III (SMA3) children.

	SMA1		SMA2		SMA3	
	median	IQR	median	IQR	median	IQR
**Anthropometry**						
n	32		51		8	
age (months)	9.8	(6.2–16.0)°	44.8	(36.4–63.8)	64.8	(54.4–79.8)
height (cm)	72.0	(66.1–85.0) °	101.8	(91.5–109.7)	120.0	(101.5–122.0)
weight (Kg)	7.6	(6.8–9.6) °	14.4	(12.2–17.0)	18.5	(15.2–21.5)
**Motor function assessment**						
n	23		34		7	
CHOP INTEND (/64)	22.0	(21.0–32.5)	-		-	
HFMSE (/66)	-		16.5	(12.0–24.9)^#^	49.0	(45.5–55.0)
ULM (/18)	-		10.0	(6.0–12.8) ^###^	18.0	(17.5–18.0)
**Spirometry and cough measurement**						
n			22		5	
FVC (L)	-		0.55	(0.47–0.89) ^###^	1.34	(1.16–1.63)
FVC (%pred)	-		69.4	(47.2–90.9)	94.3	(89.5–102.2)
FEV_1_ (L)	-		0.53	(0.40–0.71) ^#^	1.06	(1.03–1.53)
FEV_1_ (%pred)	-		61.5	(50.7–82.9)	103.7	(91.8–105.3)
FEV_1_/FVC (%)	-		89.6	(81.7–98.5)	93.0	(90.3–96.0)
PCF (L/min)	-		97.2	(59.85–112.2) ^#^	154.5	(116.1–216.2)
PCF (%pred)	-		59.8	(43.5–70.0) ^#^	73.3	(63.8–89.2)

°: p<0.05 vs SMA2,SMA3 and healthy children

#, ###: p<0.05, 0.001 vs SMA3.

SMA2 children showed reduced absolute spirometric and PCF values compared to SMA3 whose PCF was lower than predicted (Table 1).

### Breathing pattern and thoraco-abdominal contribution during QB

**Fig 3:**
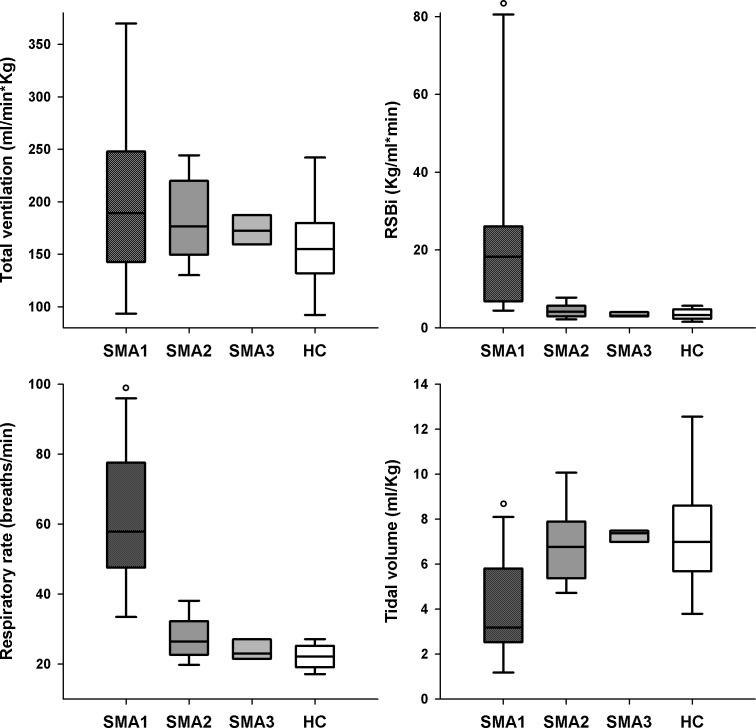
Box-and-whisker plot representing the median (line within the box), the IQR (length of the box), the 90^th^ and the 10^th^ percentiles (whiskers above and below the box) of total ventilation (V´_E_, top left panel), rapid and shallow breathing index (RSBi, top right panel), respiratory rate (bottom left panel) and tidal volume (V_T_, bottom right panel) in SMA types I, II, III and healthy children (SMA1, SMA2, SMA3 and HC, respectively) at rest during spontaneous quiet breathing in supine position. °: p<0.05 vs SMA2, SMA3 and HC

**Fig 4:**
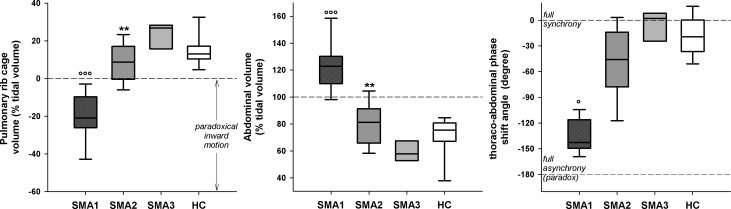
Box-and-whisker plot representing the median (line within the box), the IQR (length of the box), the 90^th^ and the 10^th^ percentiles (whiskers above and below the box) of pulmonary ribcage percentage contribution to tidal volume (left panel), abdominal percentage contribution to tidal volume (middle panel) and thoraco-abdominal phase shift angle (right panel) in SMA types I, II, III and healthy children (SMA1, SMA2, SMA3 and HC, respectively) at rest during spontaneous quiet breathing in supine position. Negative values of pulmonary ribcage percentage contribution to tidal volume indicate paradoxical inward movement of the compartment during inspiration. Phase shift angle varies between 0, when the two compartments are fully in phase, and -180° when one compartment is completely out of phase respect the other with consequent paradoxical movement. °,°°°: p<0.05, 0.001 vs SMA2, SMA3 and HC; **: p<0.01 vs SMA3

SMA1 children were characterized by normal V_E_, greater RSBi and RR, reduced V_T_ (Fig 3) with negative %ΔV_RC,P_ compensated by higher %ΔV_AB_ leading to paradoxical thoraco-abdominal motion close to complete out of phase (Fig 4). They also showed lower (p<0.001) values of %ΔV_RC,A_ (median: -2.1%; IQR: -8.7–3.2) compared to SMA2 (12.0%; 8.1–15.7), SMA3 (18.1%; 16.6–19.1) and HC (12.2%; 8.6–15.8).

**Fig 5:**
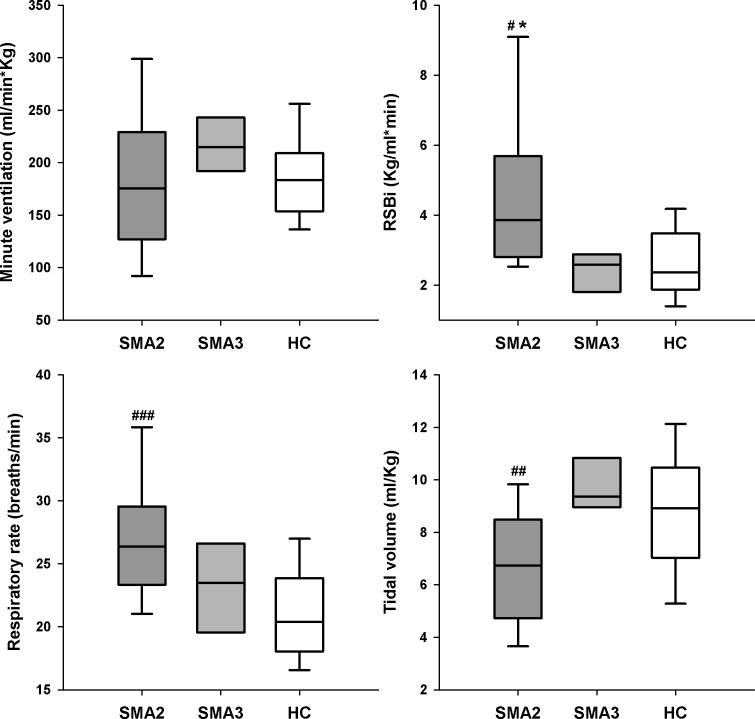
Box-and-whisker plot representing the median (line within the box), the IQR (length of the box), the 90^th^ and the 10^th^ percentiles (whiskers above and below the box) of minute ventilation (V´_E_, top left panel), rapid and shallow breathing index (RSBi, top right panel), respiratory rate (bottom left panel) and tidal volume (V_T_, bottom right panel) in SMA types II, III and healthy children (SMA2, SMA3 and HC, respectively) at rest during spontaneous quiet breathing in seated position. *: p<0.05 vs SMA3; #, ##, ###: p<0.05, 0.01, 0.001 vs HC

**Fig 6:**
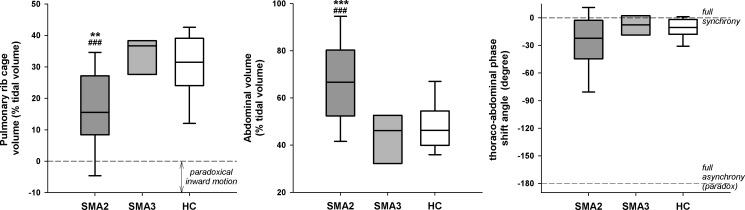
Box-and-whisker plot representing the median (line within the box), the IQR (length of the box), the 90^th^ and the 10^th^ percentiles (whiskers above and below the box) of pulmonary ribcage percentage contribution to tidal volume (left panel), abdominal percentage contribution to tidal volume (middle panel) and thoraco-abdominal phase shift angle (right panel) in SMA types II, III and healthy children (SMA2, SMA3 and HC, respectively) at rest during spontaneous quiet breathing in seated position. **,***: p<0.01, 0.001 vs SMA3; ###: p<0.001 vs HC

Lying supine, SMA2 breathed like healthy peers whereas differences emerged when seated, with normal V´_E_, higher RR and reduced V_T_ resulting in rapid and shallowed breathing (Fig 5). Despite the increased %ΔV_AB_, V_T_ was lower because of the reduced %ΔV_RC,P_ (Fig 6) with %ΔV_RC,A_ not differing among SMA2 (15.6%; 12.2–18.5), SMA3 (21.0; 17.0–28.1) and HC (20.3%; 16.7–23.5).

%ΔV_RC,P_ was negative in 14 and 6 SMA2 children, respectively in supine and seated position.

No differences were found between SMA3 and HC children.

**Fig 7:**
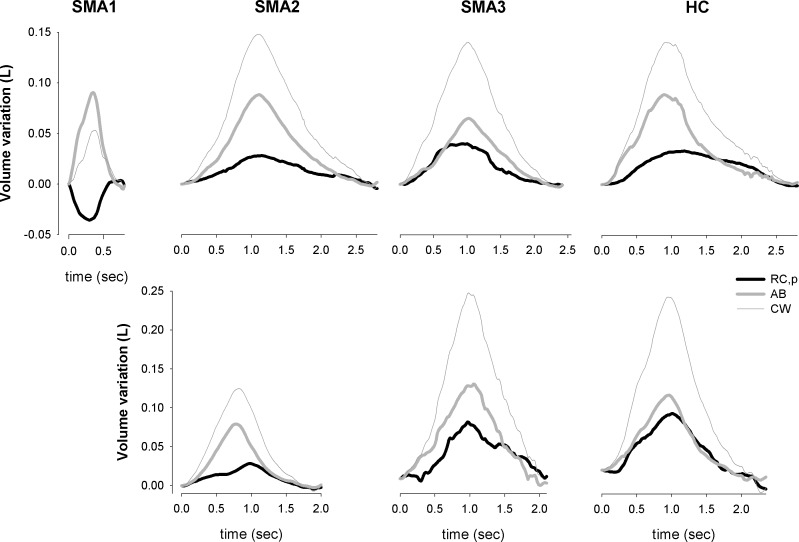
Representative traces of chest wall (CW, thin grey line), pulmonary ribcage (RC,p, black line) and abdominal (AB, bold grey line) volume variations during spontaneous quiet breathing in one represented subject belonging to SMA types I, II, III and healthy children (SMA1,SMA2, SMA3 and HC, respectively). In supine position (upper panels), the reduced tidal volume, the increased respiratory rate and the inspiratory paradoxical indrawing of the pulmonary ribcage compensated by increasing abdominal contribution in SMA1 are evident. Only in seated position (lower panels), in SMA2 total and compartmental volume change are reduced.

Fig 7 shows representative thoraco-abdominal volume traces for each considered group.

### Effect of salbutamol therapy

In SMA2, HMFSE, ULM, FVC, FEV_1_, PCF, breathing pattern and thoraco-abdominal contribution during QB in seated and supine position did not differ (p>0.05) between children on salbutamol at a stable dosage from at least 12 months and untreated children.

### Correlation between motor and respiratory function assessments

While thoraco-abdominal measurements linearly correlated with CHOP INTEND, HFMSE and ULM scales, the latter two also correlated with FVC, FEV_1_ and PCF and V_T_/Kg (Table 2).

**Table 2:** Linear regression parameters between CHOP INTEND, HFMSE and ULM scales and the respiratory function parameters significantly affected in SMA types I and II-III, respectively.

	CHOP INTEND		HFMSE		ULM	
	r^2^	p	r^2^	p	r^2^	p
**FVC (L)**			0.530	**<0.001**	0.599	**<0.001**
**FEV**_**1**_ **(L)**			0.516	**<0.001**	0.568	**<0.001**
**PCF (%pred)**			0.229	0.084	0.128	0.231
**PCF (L)**			0.328	**0.008**	0.323	**0.011**
**RR**^**§**^	0.003	0.796	0.011	0.529	0.052	0.195
**RSBi/Kg**^**§**^	0.007	0.160	0.041	0.228	0.084	0.092
**V**_**T**_**/Kg**^**§**^	0.000	0.966	0.165	**0.012**	0.204	**0.007**
**ϕ**_**TA**_^**§**^	0.217	**0.007**	0.441	**<0.001**	0.431	**<0.001**
Δ**V**_**RC,P**_ **(%V**_**T**_**)** ^**§**^	0.168	**0.020**	0.419	**<0.001**	0.407	**<0.001**
Δ**V**_**AB**_ **(%V**_**T**_**)** ^**§**^	0.219	**0.007**	0.530	**<0.001**	0.599	**<0.001**

^§^ acquired in supine position when correlated with CHOP INTEND and in seated position when correlated with HFMSE and ULM

The correlation coefficient (r^2^) quantifies the strength of the association between the variables, when p>0.05 there is no significant relationship between the two variables.

### SMA1 infants on natural history

The age range of the SMA1 group on natural history (NH) was between 4 and 12 months. In order to avoid bias due to age, and therefore to the evolution of the disease, 9 patients receiving respiratory support were excluded because older than 12 months. The remaining 13 infants receiving respiratory support showed significantly lower RR compared to the group on natural history (IQR: 50–66 and 58–84, respectively, p = 0.041).

## Discussion

In the present study the detailed description of the respiratory function in a large cohort of young (<8 yrs), and therefore hardly cooperative, children affected by the three forms of SMA is provided and compared. The assessment of the breathing pattern at rest was feasible, well tolerated and significantly different according to the severity of the disease. Our findings show that 1) SMA1 and SMA2 are characterized by weakened inspiratory ribcage muscles and spared diaphragm since childhood, as confirmed by accurate measurements of percentage contribution of rib cage and abdomen to tidal volume; 2) when evaluating respiratory muscles action, posture plays a crucial role, with ribcage muscle weakness of SMA2 emerging only in the seated position; 3) a 12-months stable dosage of salbutamol does not affect motor and respiratory functions in our cohort of SMA2; 4) in children with SMA, motor function, assessed by different disease-specific functional scales, is linearly correlated with several spirometric and respiratory function parameters during spontaneous QB; and 5) SMA1 children for whom the spontaneous course of the disease was chosen were more tachypneic compared to those receiving respiratory support.

In these children, respiratory involvement differs according to the severity of the disease, therefore, it is important to differentiate the respiratory treatment since early childhood.

*SMA type I–*Without any postural support, SMA1 patients can only adopt the supine position. In this situation, without any ventilatory assistance, while awake and in stable condition, SMA1 children are able to maintain normal levels of V´_E_ only by adopting a rapid and shallow breathing pattern, mostly due to high RR with a slightly reduced V_T_. Since RR is known to inversely correlate with age, being its variability larger in the first months of life, it is important to understand whether the high rate of RR observed in SMA1 is either a consequence of the disease or of the youngest age. Reported reference values for RR in healthy children range from 47 breath/min respectively from 2 to 36 months of age[55–57], whereas in our population of SMA1 IQR was 48–75 (Fig 2). Tachypnea, therefore, seems to be a clinical sign in SMA1, even in the absence of respiratory infection, representing the ventilatory strategy adopted by these children to compensate for their shallow breathing, in order to guarantee normal ventilation. The reduced V_T_, in turn, is due to the inspiratory paradoxical inward movement of pulmonary ribcage (IPIM_RC,p).

Under physiological conditions, in supine position the diaphragm adopts a more favorable geometry, being lengthened by the abdominal content[58–60]. As a consequence, the pressure developed and the resulting abdominal displacement is greater compared to the seated position, even with similar levels of neural drive. This is the reason why in supine position abdominal contribution to tidal volume becomes greater than ribcage[48,61], with the latter not showing any paradox thanks to the action of the iRCM. On the other hand, we observe a consistent presence of IPIM_RCp in SMA1, suggesting that iRCM are presumably not able to contrast the negative pleural pressure swings generated by diaphragmatic contraction. The presence of IPIM_RCp has at least two negative consequences. Firstly, a portion of the lung does not expand properly during inspiration. Secondly, part of diaphragm’s work is wasted to distort the chest wall rather than to inflate the lungs.

*SMA type II—*The breathing pattern of SMA2 is normal in supine, whereas differences emerge in seated position with a rapid and shallow breathing, adopted to guarantee normal ventilation, characterized by reduced %ΔV_RC,P_.

Under physiological conditions, the dependence of V_T_ and thoraco-abdominal pattern on posture is due to its effect on ribcage and abdominal compliances. Compared to supine, in seated position the former increases while the latter decreases because of the tonic activity of abdominal muscles to stabilize the trunk[62]. As a consequence, diaphragm’s contraction results more into expanding the inferior ribcage, than in moving the abdominal viscera[63] and the expansion of ΔΔ_RC,P_ becomes predominantly due to the contraction of iRCM[64,65]. The reduced %ΔΔ_RC,P_, therefore, suggests that weakened iRCM are not able to fully counterbalance the decrease of pleural pressure determined by the contraction of a preserved diaphragm, also of SMA2, leading to IPIM_RCp in some cases (12% in seated, 27% in supine) and presumably to the poor spirometry.

*SMA type III–*In our cohort of SMA3, FVC and FEV_1_ were within predicted values, with slightly lower values of PCF, and normal ventilatory and thoraco-abdominal patterns. Because of the small number in this subgroup, it was not possible to distinguish between the subtypes IIIA and IIIB, who were reported to be characterized by different respiratory features[37].

Taken together, our results confirm the general consensus that, according to the severity of the disease, SMA causes iRCM weakness with a preserved diaphragm, with the following consequences[19,23]: 1) alveolar hypoventilation, 2) poor airway clearance due to reduced cough efficacy, 3) micro-atelectasis reducing lung compliance, 4) bell-shaped chest with sternal depression reducing chest wall compliance, and 5) diaphragmatic fatigue secondary to the increased mechanical load.

%ΔV_RC,P_, therefore, represents a potential useful clinical outcome in SMA, that can be obtained by OEP in all children regardless collaboration, severity level and in different postures. OEP may be particularly useful in monitoring the progression of the disease and the effects of possible interventions and/or of new medicinal products in young SMA children. Conversely, active effort-dependent volume measurements, like spirometry and cough, have two intrinsic problems for children: 1) poor availability of reliable values due to the lack of collaboration, and 2) absence of predicted values of children younger than the age of 4 years.

Our study has some limitations: 1) absence of infants in the control group to be compared with SMA1, but normalizing V_T_ according to body weight and correcting for the total anatomical dead space allowed to take into account the difference in body size that was shown to remain invariant with age[19]; 2) reduced number of available motor function, spirometry and PCF records due either to the young age of patients or to the fact that they were not acquired close to QB analysis; 3) use of a new model of markers, that anyway provided ventilatory parameters comparable with those previously measured by pneumotachography in SMA children[19,23,56,57,66,67].

The strength of this study is that the analysis was performed in a large cohort of young SMA children and controls during awake QB, in the positions commonly adopted in daily life, without any specific requested maneuver. The study has also clinical implications and demonstrated: 1) the importance of posture when evaluating respiratory muscle function; 2) the need for larger prospective randomized, double-blind, placebo controlled trials to better understand the potentiality of salbutamol on motor and respiratory functions in SMA patients (in contrast with other works[34–36] we did not find any difference on motor and respiratory function in SMA2-treated children); 3) the potentiality of CHOP INTEND, HFMSE and ULM scales to predict poor respiratory outcomes, in particular the inefficient action of iRCM, although they do not specifically assess any aspect of respiratory function; and 4) the effect of proactive respiratory assistance in SMA1 infants to help reducing tachypnea. It was not possible to carry out a similar comparison for SMA2 because in these children the respiratory management was performed according to the international guidelines[11,39], and proactive and multidisciplinary management was adopted in all patients. We believe that such respiratory care and assistance may be the reason of the relatively limited incidence of hospitalization in SMA2 children.

## Conclusions

A negative or reduced %ΔV_RC,P_, indicative of ribcage muscle weakness, is a distinctive feature of SMA1 and SMA2 since infancy. Its quantitative assessment represents a non-invasive, non-volitional index that can be used to detect changes on the respiratory function over time and the effects of specific interventions and/or of clinical pharmacological trials in all forms of SMA. Low values on SMA-specific motor function scales indicate impairment not only of motor but also of respiratory functions.

## Abbreviations

ANOVA: Analysis of Variance
CHOP INTEND: Children’s Hospital of Philadelphia Infant Test for Neuromuscular Disorders scale
FEV_1_: Forced Expiratory Volume in one second
FVC: Forced Vital Capacity
HC: Healthy Children
HFMSE: Hammersmith Functional Motor Scale Expanded
IPIM_RCp: Inward Paradoxical Inspiratory Movement of Pulmonary Rib Cage
IQR: Interquartile Range
iRCM: inspiratory ribcage muscles
OEP: Opto-Electronic Plethysmography
PCF: Peak Cough Flow
QB: spontaneous Quiet Breathing at rest
RR: respiratory rate
RSBi: Rapid and Shallow Breathing index
SMA: Spinal Muscular Atrophy
SMA1: SMA type I
SMA2: SMA type II
SMA3: SMA type III
ULM: Upper Limb Module
VTAp: the ventilatory and thoraco-abdominal pattern
V’_E_: minute ventilation
V_T_: tidal volume
ΔV_AB_: volume variations of the abdomen
ΔV_CW_: volume variations of the chest wall
ΔV_RC,A_: volume variations of the abdominal ribcage
ΔV_RC,P_: volume variations of the pulmonary ribcage
ϕ_TA_: thoraco-abdominal phase shift angle between pulmonary ribcage and abdomen
%ΔV_AB_: abdominal percentage contribution to tidal volume
%ΔV_RC,A_: abdominal ribcage percentage contribution to tidal volume
%ΔV_RC,P_: pulmonary ribcage percentage contribution to tidal volume

## References

[1] Mercuri E, Bertini E. Stem cells in severe infantile spinal muscular atrophy. Neuromuscul Disord [Internet]. 2012 12 [cited 2015 Jul 2];22(12):1105. 10.1016/j.nmd.2012.11.001 23206850

[2] Ogino S, Wilson RB. Genetic testing and risk assessment for spinal muscular atrophy (SMA). Hum Genet [Internet]. 2002 12 [cited 2015 Jul 20];111(6):477–500. 10.1007/s00439-002-0828-x 12436240

[3] Ogino S, Wilson RB, Gold B. New insights on the evolution of the SMN1 and SMN2 region: simulation and meta-analysis for allele and haplotype frequency calculations. Eur J Hum Genet [Internet]. 2004 12 [cited 2015 Jul 20];12(12):1015–23. 10.1038/sj.ejhg.5201288 15470363

[4] Darras BT. Spinal muscular atrophies. Pediatr Clin North Am [Internet]. 2015 6 [cited 2016 Apr 17];62(3):743–66. 10.1016/j.pcl.2015.03.010 26022173

[5] Van Meerbeke JP, Sumner CJ. Progress and promise: the current status of spinal muscular atrophy therapeutics. Discov Med [Internet]. 2011 10 [cited 2016 Jun 1];12(65):291–305. 22031667

[6] Mercuri E, Finkel R, Montes J, Mazzone ES, Sormani MP, Main M, et al Patterns of disease progression in type 2 and 3 SMA: Implications for clinical trials. Neuromuscul Disord [Internet]. 2016 2 [cited 2016 May 25];26(2):126–31. 10.1016/j.nmd.2015.10.006 26776503 PMC4762230

[7] Dubowitz V. Ramblings in the history of spinal muscular atrophy. Neuromuscul Disord [Internet]. 2009 1 [cited 2016 May 25];19(1):69–73. 10.1016/j.nmd.2008.10.004 18951794

[8] Dubowitz V. Chaos in the classification of SMA: a possible resolution. Neuromuscul Disord [Internet]. 1995 1 [cited 2016 May 25];5(1):3–5. 7719138 10.1016/0960-8966(94)00075-k

[9] Wang CH, Finkel RS, Bertini ES, Schroth M, Simonds A, Wong B, et al Consensus statement for standard of care in spinal muscular atrophy. J Child Neurol [Internet]. 2007 8 [cited 2015 Jun 12];22(8):1027–49. 10.1177/0883073807305788 17761659

[10] Schroth MK. Special considerations in the respiratory management of spinal muscular atrophy. Pediatrics [Internet]. 2009 5 [cited 2015 Nov 18];123 Suppl:S245–9. 10.1542/peds.2008-2952K 19420154

[11] Sansone VA, Racca F, Ottonello G, Vianello A, Berardinelli A, Crescimanno G, et al 1st Italian SMA Family Association Consensus Meeting:: Management and recommendations for respiratory involvement in spinal muscular atrophy (SMA) types I-III, Rome, Italy, 30–31 January 2015. Neuromuscul Disord [Internet]. 2015 Sep 18 [cited 2015 Nov 18]; 10.1016/j.nmd.2015.09.009 26453142

[12] Bach JR. The use of mechanical ventilation is appropriate in children with genetically proven spinal muscular atrophy type 1: the motion for. Paediatr Respir Rev [Internet]. 2008 3 [cited 2015 Dec 3];9(1):45-50-6. 10.1016/j.prrv.2007.11.003 18280979

[13] Bach JR, Saltstein K, Sinquee D, Weaver B, Komaroff E. Long-term survival in Werdnig-Hoffmann disease. Am J Phys Med Rehabil [Internet]. 2007 5 [cited 2016 Apr 17];86(5):339-45-8, 379. 10.1097/PHM.0b013e31804a8505 17449977

[14] Petrone A, Pavone M, Testa MBC, Petreschi F, Bertini E, Cutrera R. Noninvasive ventilation in children with spinal muscular atrophy types 1 and 2. Am J Phys Med Rehabil [Internet]. 2007 3 [cited 2015 Jun 25];86(3):216–21. 10.1097/PHM.0b013e31802ef774 17314706

[15] Finkel R, Bertini E, Muntoni F, Mercuri E. 209th ENMC International Workshop: Outcome Measures and Clinical Trial Readiness in Spinal Muscular Atrophy 7–9 November 2014, Heemskerk, The Netherlands. Neuromuscul Disord [Internet]. 2015 7 [cited 2016 Apr 4];25(7):593–602. 10.1016/j.nmd.2015.04.009 26045156

[16] Lewelt A, Newcomb TM, Swoboda KJ. New therapeutic approaches to spinal muscular atrophy. Curr Neurol Neurosci Rep [Internet]. 2012 2 [cited 2016 Jun 1];12(1):42–53. 10.1007/s11910-011-0240-9 22134788 PMC3260050

[17] Perez A, Mulot R, Vardon G, Barois A, Gallego J. Thoracoabdominal pattern of breathing in neuromuscular disorders. Chest [Internet]. 1996 8 [cited 2015 Jun 24];110(2):454–61. 8697851 10.1378/chest.110.2.454

[18] Lissoni A, Aliverti A, Molteni F, Bach JR. Spinal muscular atrophy: kinematic breathing analysis. Am J Phys Med Rehabil [Internet]. 1 [cited 2015 Jul 7];75(5):332–9. 8873699 10.1097/00002060-199609000-00005

[19] Finkel RS, Weiner DJ, Mayer OH, Mcdonough JM, Panitch HB. Respiratory muscle function in infants with spinal muscular atrophy type I. Pediatr Pulmonol. 2014;1242(August 2013):1234–42. 10.1002/ppul.22997 24777943

[20] Nicot F, Hart N, Forin V, Boulé M, Clément A, Polkey MI, et al Respiratory muscle testing: a valuable tool for children with neuromuscular disorders. Am J Respir Crit Care Med [Internet]. 2006 7 1 [cited 2015 May 28];174(1):67–74. 10.1164/rccm.200512-1841OC 16574932

[21] Fauroux B, Quijano-Roy S, Desguerre I, Khirani S. The value of respiratory muscle testing in children with neuromuscular disease. Chest [Internet]. 2015 2 [cited 2015 Apr 27];147(2):552–9. 10.1378/chest.14-0819 25644908

[22] Bach JR, Saporito LR. Criteria for extubation and tracheostomy tube removal for patients with ventilatory failure. A different approach to weaning. Chest [Internet]. 1996 12 [cited 2015 May 28];110(6):1566–71. 8989078 10.1378/chest.110.6.1566

[23] Khirani S, Colella M, Caldarelli V, Aubertin G, Boulé M, Forin V, et al Longitudinal course of lung function and respiratory muscle strength in spinal muscular atrophy type 2 and 3. Eur J Paediatr Neurol [Internet]. 2013 11 [cited 2015 May 26];17(6):552–60. 10.1016/j.ejpn.2013.04.004 23672834

[24] Kaufmann P, McDermott MP, Darras BT, Finkel R, Kang P, Oskoui M, et al Observational study of spinal muscular atrophy type 2 and 3: functional outcomes over 1 year. Arch Neurol [Internet]. 2011 6 [cited 2015 Jun 16];68(6):779–86. 10.1001/archneurol.2010.373 21320981 PMC3839315

[25] Fujak A, Raab W, Schuh A, Richter S, Forst R, Forst J. Natural course of scoliosis in proximal spinal muscular atrophy type II and IIIa: descriptive clinical study with retrospective data collection of 126 patients. BMC Musculoskelet Disord [Internet]. 2013 1 [cited 2015 Jun 16];14:283 10.1186/1471-2474-14-283 24093531 PMC3850509

[26] Chng SY, Wong YQ, Hui JH, Wong HK, Ong HT, Goh DY. Pulmonary function and scoliosis in children with spinal muscular atrophy types II and III. J Paediatr Child Health [Internet]. 2003 12 [cited 2015 Jun 16];39(9):673–6. 14629498 10.1046/j.1440-1754.2003.00266.x

[27] Ioos C, Leclair-Richard D, Mrad S, Barois A, Estournet-Mathiaud B. Respiratory capacity course in patients with infantile spinal muscular atrophy. Chest [Internet]. 2004 9 [cited 2015 Nov 18];126(3):831–7. 10.1378/chest.126.3.831 15364763

[28] Bach JR, Tuccio MC, Khan U, Saporito LR. Vital capacity in spinal muscular atrophy. Am J Phys Med Rehabil [Internet]. 2012 6 [cited 2015 Nov 20];91(6):487–93. 10.1097/PHM.0b013e31824fa5dd 22469873

[29] Gormley MC. Respiratory management of spinal muscular atrophy type 2. J Neurosci Nurs [Internet]. 2014 12 [cited 2016 May 31];46(6):E33–41. 10.1097/JNN.0000000000000080 25365058

[30] Miske LJ, Hickey EM, Kolb SM, Weiner DJ, Panitch HB. Use of the mechanical in-exsufflator in pediatric patients with neuromuscular disease and impaired cough. Chest [Internet]. 2004 4 [cited 2016 May 8];125(4):1406–12. 15078753 10.1378/chest.125.4.1406

[31] Fauroux B, Guillemot N, Aubertin G, Nathan N, Labit A, Clément A, et al Physiologic benefits of mechanical insufflation-exsufflation in children with neuromuscular diseases. Chest [Internet]. 2008 1 [cited 2016 Apr 17];133(1):161–8. 10.1378/chest.07-1615 18071020

[32] Kuzuhara S, Chou SM. Preservation of the phrenic motoneurons in Werdnig-Hoffmann disease. Ann Neurol [Internet]. 1981 5 [cited 2016 Jan 7];9(5):506–10. 10.1002/ana.410090515 7271245

[33] Macklem PT. Respiratory muscles: the vital pump. Chest [Internet]. 1980 11 [cited 2015 Oct 21];78(5):753–8. 7428459 10.1378/chest.78.5.753

[34] Kinali M, Mercuri E, Main M, De Biasia F, Karatza A, Higgins R, et al Pilot trial of albuterol in spinal muscular atrophy. Neurology [Internet]. 2002 8 27 [cited 2015 Nov 16];59(4):609–10. 12196659 10.1212/wnl.59.4.609

[35] Pane M, Staccioli S, Messina S, D’Amico A, Pelliccioni M, Mazzone ES, et al Daily salbutamol in young patients with SMA type II. Neuromuscul Disord [Internet]. 2008 7 [cited 2015 Jul 13];18(7):536–40. 10.1016/j.nmd.2008.05.004 18579379

[36] Tiziano FD, Lomastro R, Pinto AM, Messina S, D’Amico A, Fiori S, et al Salbutamol increases survival motor neuron (SMN) transcript levels in leucocytes of spinal muscular atrophy (SMA) patients: relevance for clinical trial design. J Med Genet [Internet]. 2010 12 [cited 2016 May 20];47(12):856–8. 10.1136/jmg.2010.080366 20837492

[37] LoMauro A, Romei M, Priori R, Laviola M, D’Angelo MG, Aliverti A. Alterations of thoraco-abdominal volumes and asynchronies in patients with spinal muscle atrophy type III. Respir Physiol Neurobiol [Internet]. 2014 6 15 [cited 2015 Oct 7];197:1–8. 10.1016/j.resp.2014.03.001 24632504

[38] Finkel RS, Weiner DJ, Mayer OH, McDonough JM, Panitch HB. Respiratory muscle function in infants with spinal muscular atrophy type I. Pediatr Pulmonol [Internet]. 2014 12 [cited 2015 Jul 8];49(12):1234–42. 10.1002/ppul.22997 24777943

[39] Wang CH, Finkel RS, Bertini ES, Schroth M, Simonds A, Wong B, et al Consensus statement for standard of care in spinal muscular atrophy. J Child Neurol [Internet]. 2007 8 [cited 2016 Jul 11];22(8):1027–49. 10.1177/0883073807305788 17761659

[40] Glanzman AM, Mazzone E, Main M, Pelliccioni M, Wood J, Swoboda KJ, et al The Children’s Hospital of Philadelphia Infant Test of Neuromuscular Disorders (CHOP INTEND): test development and reliability. Neuromuscul Disord [Internet]. 2010 3 [cited 2015 Jul 13];20(3):155–61. 10.1016/j.nmd.2009.11.014 20074952 PMC3260046

[41] Glanzman AM, McDermott MP, Montes J, Martens WB, Flickinger J, Riley S, et al Validation of the Children’s Hospital of Philadelphia Infant Test of Neuromuscular Disorders (CHOP INTEND). Pediatr Phys Ther [Internet]. 2011 1 [cited 2015 Jul 4];23(4):322–6. 10.1097/PEP.0b013e3182351f04 22090068

[42] Mazzone E, Bianco F, Martinelli D, Glanzman AM, Messina S, Sanctis R De, et al Assessing upper limb function in nonambulant SMA patients: Development of a new module. Neuromuscul Disord [Internet]. 2011 6 [cited 2015 Jul 2];21(6):406–12. 10.1016/j.nmd.2011.02.014 21421316

[43] Beydon N, Davis SD, Lombardi E, Allen JL, Arets HGM, Aurora P, et al An official American Thoracic Society/European Respiratory Society statement: pulmonary function testing in preschool children. Am J Respir Crit Care Med [Internet]. 2007 6 15 [cited 2016 Oct 4];175(12):1304–45. 10.1164/rccm.200605-642ST 17545458

[44] Quanjer PH, Hall GL, Stanojevic S, Cole TJ, Stocks J. Age- and height-based prediction bias in spirometry reference equations. Eur Respir J. 2012;40(1):190–7. 10.1183/09031936.00161011 22183491

[45] Quanjer PH, Stanojevic S, Cole TJ, Baur X, Hall GL, Culver BH, et al Multi-ethnic reference values for spirometry for the 3-95-yr age range: the global lung function 2012 equations. Eur Respir J [Internet]. 2012 12 [cited 2015 Apr 2];40(6):1324–43. 10.1183/09031936.00080312 22743675 PMC3786581

[46] Bianchi C, Baiardi P. Cough peak flows: standard values for children and adolescents. Am J Phys Med Rehabil [Internet]. 2008 6 [cited 2015 Jun 15];87(6):461–7. 10.1097/PHM.0b013e318174e4c7 18496248

[47] Cala SJ, Kenyon CM, Ferrigno G, Carnevali P, Aliverti A, Pedotti A, et al Chest wall and lung volume estimation by optical reflectance motion analysis. J Appl Physiol [Internet]. 1996 12 [cited 2015 Jun 15];81(6):2680–9. 9018522 10.1152/jappl.1996.81.6.2680

[48] Romei M, Mauro A Lo, D’Angelo MG, Turconi AC, Bresolin N, Pedotti A, et al Effects of gender and posture on thoraco-abdominal kinematics during quiet breathing in healthy adults. Respir Physiol Neurobiol [Internet]. 2010 7 31 [cited 2015 May 17];172(3):184–91. 10.1016/j.resp.2010.05.018 20510388

[49] Nugent JK. The Brazelton Neonatal Behavioral Assessment Scale: implications for intervention. Pediatr Nurs [Internet]. [cited 2016 Oct 4];7(3):18–21, 67. 6909673

[50] Numa AH, Newth CJ. Anatomic dead space in infants and children. J Appl Physiol [Internet]. 1996 5 [cited 2015 Nov 27];80(5):1485–9. 8727530 10.1152/jappl.1996.80.5.1485

[51] Aliverti A, Dellacà R, Pelosi P, Chiumello D, Gatihnoni L, Pedoti A. Compartmental analysis of breathing in the supine and prone positions by optoelectronic plethysmography. Ann Biomed Eng [Internet]. 2001 1 [cited 2015 Jun 15];29(1):60–70. 11219508 10.1114/1.1332084

[52] Dellaca’ RL, Ventura ML, Zannin E, Natile M, Pedotti A TP. Measurement of total and compartmental lung volume changes in newborns by optoelectronic plethysmography. Pediatr Res. 2010;67((1)):11–6. 10.1203/PDR.0b013e3181c0b184 19755932

[53] Reinaux CMA, Aliverti A, da Silva LGM, da Silva RJ, Gonçalves JN, Noronha JB, et al Tidal volume measurements in infants: Opto-electronic plethysmography versus pneumotachograph. Pediatr Pulmonol [Internet]. 2016 3 17 [cited 2016 May 19]; 10.1002/ppul.23394 26991671

[54] Vieira DS, Hoffman M, Pereira DA, Britto RR PV. Optoelectronic plethysmography: intra-rater and inter-rater reliability in healthy subjects. Respir Physiol Neurobiol [Internet]. 2013;189(3):473–6. 10.1016/j.resp.2013.08.023 24036178

[55] Berman S, Simoes EA, Lanata C. Respiratory rate and pneumonia in infancy. Arch Dis Child [Internet]. 1991 1 [cited 2015 Oct 6];66(1):81–4. 1994857 10.1136/adc.66.1.81 PMC1793190

[56] Simoes EA, Roark R, Berman S, Esler LL, Murphy J. Respiratory rate: measurement of variability over time and accuracy at different counting periods. Arch Dis Child [Internet]. 1991 10 [cited 2015 Oct 6];66(10):1199–203. 1953002 10.1136/adc.66.10.1199 PMC1793530

[57] Rusconi F, Castagneto M, Gagliardi L, Leo G, Pellegatta A, Porta N, et al Reference values for respiratory rate in the first 3 years of life. Pediatrics [Internet]. 1994 9 [cited 2015 Oct 6];94(3):350–5. 8065862

[58] Leduc D, De Troyer A. Function of the canine inspiratory muscle pump in pleural effusion: influence of body position. J Appl Physiol [Internet]. 2013 4 [cited 2016 May 25];114(7):941–7. 10.1152/japplphysiol.01392.2012 23393063

[59] Bushby K, Muntoni F, Urtizberea A, Hughes R, Griggs R. Report on the 124th ENMC International Workshop. Treatment of Duchenne muscular dystrophy; defining the gold standards of management in the use of corticosteroids. 2–4 April 2004, Naarden, The Netherlands. Neuromuscul Disord [Internet]. 2004 Sep [cited 2015 Jun 10];14(8–9):526–34. 10.1016/j.nmd.2004.05.006 15336694

[60] Takazakura R, Takahashi M, Nitta N, Murata K. Diaphragmatic motion in the sitting and supine positions: Healthy subject study using a vertically open magnetic resonance system. J Magn Reson Imaging [Internet]. 2004 5 [cited 2016 May 25];19(5):605–9. 10.1002/jmri.20051 15112310

[61] Lumb AB, Nunn JF. Respiratory function and ribcage contribution to ventilation in body positions commonly used during anesthesia. Anesth Analg [Internet]. 1991 10 [cited 2016 Jun 1];73(4):422–6. 1897767 10.1213/00000539-199110000-00010

[62] Mayer OH, Clayton RG, Jawad AF, McDonough JM, Allen JL. Respiratory inductance plethysmography in healthy 3- to 5-year-old children. Chest [Internet]. 2003 11 [cited 2015 Oct 6];124(5):1812–9. 14605053 10.1378/chest.124.5.1812

[63] De Troyer A. Respiratory effect of the lower rib displacement produced by the diaphragm. J Appl Physiol [Internet]. 2012 2 [cited 2016 May 25];112(4):529–34. 10.1152/japplphysiol.01067.2011 22134697

[64] De Troyer A, Kelly S, Macklem PT, Zin WA. Mechanics of intercostal space and actions of external and internal intercostal muscles. J Clin Invest [Internet]. 1985 3 [cited 2015 Jun 19];75(3):850–7. 10.1172/JCI111782 3980728 PMC423614

[65] De Troyer A, Kirkwood PA, Wilson TA. Respiratory action of the intercostal muscles. Physiol Rev [Internet]. 2005 4 [cited 2016 May 14];85(2):717–56. 10.1152/physrev.00007.2004 15788709

[66] Gaultier C, Perret L, Boule M, Buvry A, Girard F. Occlusion pressure and breathing pattern in healthy children. Respir Physiol [Internet]. 1981 10 [cited 2015 Oct 7];46(1):71–80. 7330494 10.1016/0034-5687(81)90069-4

[67] Tangsrud SE, Carlsen KC, Lund-Petersen I, Carlsen KH. Lung function measurements in young children with spinal muscle atrophy; a cross sectional survey on the effect of position and bracing. Arch Dis Child. 2001;84(6):521–4. 10.1136/adc.84.6.521 11369575 PMC1718814

